# Clinical Presentation, Management, and Outcomes of Retroperitoneal Fibrosis: A 10-Year Single-Center Study of 24 Cases

**DOI:** 10.7759/cureus.114803

**Published:** 2026-08-19

**Authors:** Anouar El Moudane, Touil Mohammed Amine, Mohammed Ramdani, Hakim El Bouanani, Achraf Benamou, Anass El Alaoui, Ali Barki

**Affiliations:** 1 Urology, Mohammed VI University Hospital, Oujda, MAR; 2 Urology, Mohammed I University, Oujda, MAR

**Keywords:** case series, corticosteroid therapy, d-j stents, retroperitoneal fibrosis, ureteral obstruction

## Abstract

Background and objective

Retroperitoneal fibrosis (RPF) is a rare fibro-inflammatory condition characterized by the formation of fibrous tissue in the retroperitoneal space. It can lead to ureteral obstruction and renal complications. Due to its nonspecific clinical presentation, diagnosis may be delayed. This study aimed to describe the clinical presentation, diagnostic findings, management strategies, and clinical outcomes of patients with RPF.

Materials and methods

We conducted a retrospective, single-center, descriptive study over 10 years, from May 2015 to May 2025, in the Department of Urology at Mohammed VI University Hospital, Oujda, Morocco. Twenty-four patients diagnosed with RPF were included.

Results

The cohort comprised 18 men (75%) and six women (25%), with a mean age of 54 years. The most common presenting symptom was low back pain, observed in 19 patients (79.2%), followed by weight loss in 17 patients (70.8%). Anuria was noted in three patients (12.5%). Acute renal failure was observed in 15 patients (62.5%). The diagnosis was established by CT in all patients, while biopsy was performed in five patients (20.8%). Idiopathic RPF was identified in 18 patients (75%). A history of retroperitoneal surgery was found in five patients (20.8%), and atherosclerosis was noted in one patient (4.2%). All patients underwent urinary drainage using a double-J stent and received systemic corticosteroid therapy. Renal function improved in six of the 15 patients presenting with acute renal failure (40%) after two months of treatment. Relapse occurred in four patients (16.7%) during a mean follow-up period of five years.

Conclusions

RPF should be considered in patients presenting with systemic symptoms and obstructive renal failure. In this case series, CT played a key role in the diagnosis, and urinary drainage combined with corticosteroid therapy was the main treatment approach. Long-term follow-up remains essential, as recurrence may occur over time.

## Introduction

Retroperitoneal fibrosis (RPF) is a rare fibro-inflammatory disorder characterized by the development of fibrous tissue within the retroperitoneum, typically surrounding the abdominal aorta and iliac vessels and often extending to entrap the ureters. Progressive ureteral involvement may result in obstructive uropathy, hydronephrosis, and renal dysfunction [1]. RPF may be classified as idiopathic or secondary. Idiopathic forms account for the majority of cases, whereas secondary RPF has been associated with medications, malignancies, infections, radiation therapy, surgery, trauma, and autoimmune disorders. Despite advances in diagnostic imaging and increased recognition of immunoglobulin G4 (IgG4)-related disease, the pathogenesis of RPF remains incompletely understood [2].

The clinical presentation is often nonspecific and may include low back pain, abdominal pain, constitutional symptoms, and manifestations related to ureteral obstruction. Consequently, diagnosis is frequently delayed until complications such as hydronephrosis or renal impairment develop [1,2]. Cross-sectional imaging, particularly contrast-enhanced CT (CECT) and MRI, plays a central role in diagnosis and in assessing disease extent [2,3]. Management strategies include emergency urinary drainage in patients presenting with obstructive uropathy and renal dysfunction, corticosteroid therapy as the cornerstone of medical treatment, and ureterolysis for refractory cases or persistent ureteral obstruction despite medical therapy. However, because of the rarity of the disease, high-quality evidence guiding the optimal therapeutic approach remains limited [4,5,6].

This retrospective, single-center study aimed to describe the clinical presentation, diagnostic findings, management strategies, and specific clinical outcomes - namely renal function recovery and disease recurrence - in patients with RPF treated in the Department of Urology at Mohammed VI University Hospital, Oujda, over 10 years.

## Materials and methods

Study design

This was a retrospective, single-center, descriptive study conducted in the Department of Urology at Mohammed VI University Hospital, Oujda, Morocco. The study included patients diagnosed with RPF over 10 years, from May 2015 to May 2025.

Study participants

All patients diagnosed with RPF during the study period were eligible for inclusion, regardless of etiology. Patients with incomplete medical records or insufficient clinical, biological, or radiological data were excluded from the analysis.

The diagnosis of RPF was established either by histopathological examination when biopsy was performed or, in the absence of histological confirmation, by characteristic radiological findings on CECT. Radiological criteria included a homogeneous retroperitoneal soft-tissue mass or plaque surrounding the abdominal aorta and/or iliac vessels, with possible extension to adjacent structures and associated ureteral encasement. For patients presenting with renal impairment at diagnosis, the imaging strategy was adapted according to clinical circumstances. CECT was performed if renal function permitted. In cases of significant impairment, CECT was delayed until after urinary drainage and renal recovery. Although MRI is part of our institutional protocol when iodinated contrast remains contraindicated [2,3], it was not required for any patient in this specific cohort, as renal function recovered sufficiently in all initially impaired patients to permit CECT. For each patient, demographic, clinical, biological, radiological, therapeutic, and follow-up data were collected from medical records.

Outcome measures

The principal clinical outcomes evaluated in this study were renal function recovery and disease recurrence. Renal recovery was defined as the normalization of serum creatinine levels to the patient's baseline or a reduction of at least 50% from the peak value at initial presentation following urinary drainage and medical therapy. Recurrence (or relapse) was defined as the reappearance of clinical symptoms, deterioration of renal function, or radiological progression of the retroperitoneal fibrotic mass during the follow-up period after an initial favorable response.

Ethical considerations

The study was conducted in accordance with the ethical principles of the Declaration of Helsinki. Patient confidentiality and anonymity were maintained throughout the study. Given the retrospective nature of the study, informed consent was waived.

Statistical analysis

Data were analyzed using IBM SPSS Statistics software (Version 27; IBM Corp., Armonk, NY). Continuous variables were expressed as means with ranges to reflect central clinical tendencies. Categorical variables, including predefined age groups, were expressed as frequencies and percentages. Categorical variables were expressed as frequencies and percentages.

## Results

Baseline characteristics

**Figure 1:**
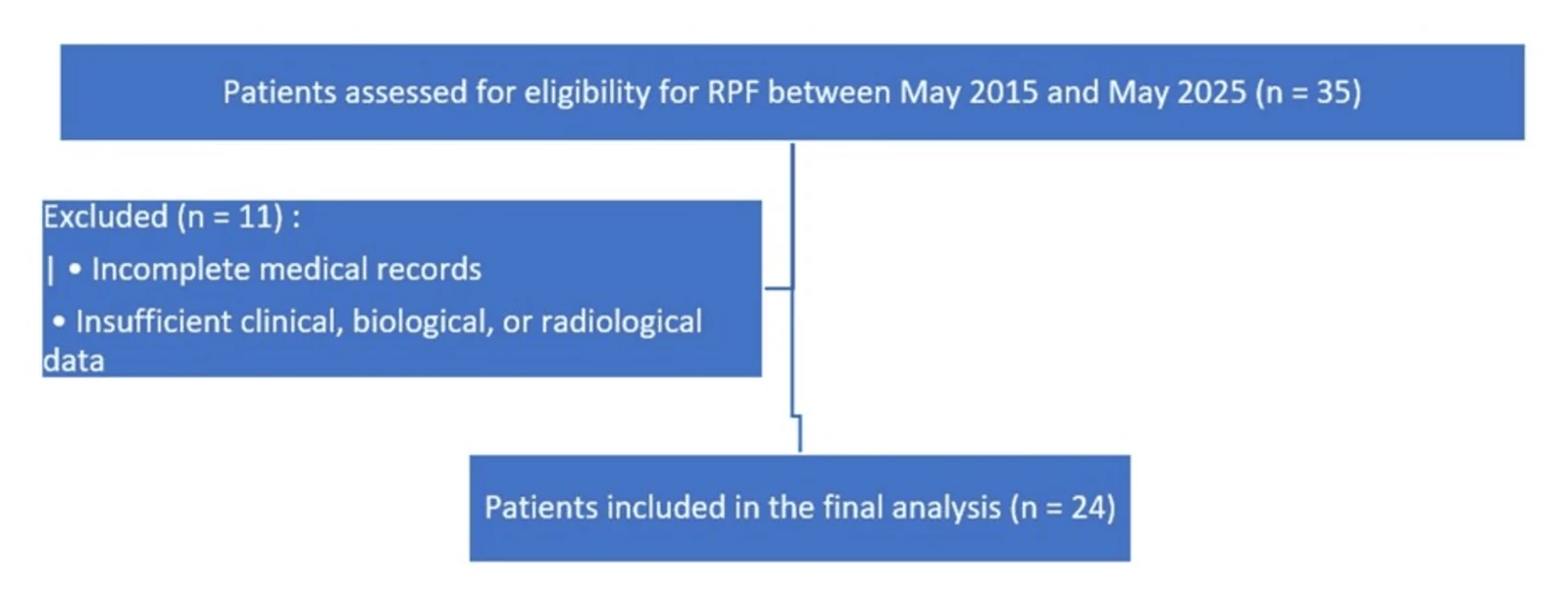
Flowchart depicting patient selection and inclusion

During the study period, after applying the exclusion criteria for incomplete medical records or insufficient data, 24 patients were included in the final analysis (Figure 1). The cohort comprised 18 men (75%) and six women (25%). The mean age at diagnosis was 54 years (range: 30-79 years). A history of chronic smoking was reported in 15 patients (62.5%), chronic medication use in 14 patients (58.3%), specifically antihypertensives in 14 patients, with concurrent oral antidiabetics in 11 patients and a statin in one patient, and previous retroperitoneal surgery in five patients (20.8%) and atherosclerosis in one patient (4.2%). The baseline characteristics of the study population are summarized in Table 1.

**Table 1:** Baseline characteristics of the study population

Variable	Value
Number of patients	24
Male sex, n (%)	18 (75%)
Female sex, n (%)	6 (25%)
Mean age, years	54
Age range, years	30–79
Age group, years, n (%)	
20–30	0 (0%)
31–40	4 (16,7%)
41–50	4 (16,7%)
51–60	7 (29,2%)
61–70	6 (25%)
> 71	3 (12,5%)
Smoking history, n (%)	15 (62.5%)
Chronic medication use, n (%)	14 (58.3%)
Previous retroperitoneal surgery, n (%)	5 (20.8%)
Atherosclerosis, n (%)	1 (4.2%)

Clinical and radiological findings

Low back pain was the most common presenting symptom, occurring in 19 patients (79.2%). Constitutional symptoms were frequent, with weight loss reported in 17 patients (70.8%) and fever in seven patients (29.2%). Anuria was observed in three patients (12.5%). Locoregional manifestations, including varicocele, hydrocele, and lower-limb edema, were present in nine patients (37.5%). An inflammatory syndrome, characterized by elevated C-reactive protein levels, was observed in all patients (100%). At initial presentation, acute renal failure secondary to ureteral obstruction was present in 15 patients (62.5%).

Ultrasonography revealed ureterohydronephrosis in all patients (100%). CECT was performed in all cases and confirmed the diagnosis of RPF associated with ureterohydronephrosis. Specifically, 14 patients underwent immediate CECT, while 10 patients had their CECT deferred after urinary drainage due to initial severe renal impairment. Biopsy of the retroperitoneal plaque was performed in five patients (20.8%). The indications for biopsy in these specific cases included atypical radiological features (such as an unusual location of the fibrotic mass or heterogeneous enhancement) and a high clinical suspicion of underlying malignancy due to severe weight loss and atypical clinical presentation. Histological examination revealed nonspecific fibrous tissue with inflammatory infiltrates in all five cases, effectively ruling out malignancy and specific infectious processes. The main clinical, biological, and radiological findings are summarized in Table 2. Idiopathic retroperitoneal fibrosis accounted for 18 cases (75%), whereas secondary forms accounted for 6 cases (25%).

**Table 2:** Clinical, biological, and radiological findings CRP: C-reactive protein; CECT: contrast-enhanced computed tomography

Finding	N (%)
Low back pain	19 (79.2%)
Weight loss	17 (70.8%)
Fever	7 (29.2%)
Anuria	3 (12.5%)
Locoregional manifestations (varicocele, hydrocele, lower-limb edema)	9 (37.5%)
Elevated CRP	24 (100%)
Acute renal failure	15 (62.5%)
Ureterohydronephrosis on ultrasound	24 (100%)
CECT-confirmed retroperitoneal fibrosis	24 (100%)
Plaque biopsy performed	5 (20.8%)

Treatment and outcomes

All patients underwent urinary drainage with double-J ureteral stent placement and received systemic corticosteroid therapy. Prednisone was initiated at a dose of 60 mg/day for one to two months, followed by gradual tapering by 10 mg per month to a maintenance dose of 5-10 mg/day. The mean treatment duration was 16 months (range: 2-24 months). Among the 15 patients who presented with acute renal failure, renal function improved in six patients (40%) after two months of treatment. Relapse occurred in four patients (16.7%) after a median follow-up period of three years (range: 12 months to five years). Seven patients (29.2%) discontinued treatment without medical advice. No deaths directly attributable to RPF or its treatment were recorded.

Three patients (12.5%) developed corticosteroid-induced diabetes mellitus and hypertension during follow-up. Treatment modalities and outcomes are summarized in Table 3. The standard institutional follow-up protocol included clinical and biochemical evaluations at one, three, and six months following the initiation of treatment, and subsequently every 6-12 months. Radiological follow-up was typically performed at three to six months to evaluate the morphological regression of the fibrotic mass.

**Table 3:** Treatment and outcomes RPF: retroperitoneal fibrosis

Variable	N (%)
Double-J stent drainage	24 (100%)
Corticosteroid therapy	24 (100%)
Renal function improvement (among patients with acute renal failure, n = 15)	6 (40%)
Relapse during follow-up	4 (16.7%)
Treatment discontinuation without medical advice	7 (29.2%)
Corticosteroid-induced diabetes and/or hypertension	3 (12.5%)
RPF-related mortality	0 (0%)

## Discussion

RPF is a rare condition, with an estimated incidence of 0.1-1.3 per 100,000 persons in the general population per year [1,6]. However, some literature suggests that it may be even less common, with reported incidence rates ranging from one per 200,000 to 500,000 persons. Despite its rarity, the condition is increasingly recognized, likely due to advancements in and greater availability of cross-sectional imaging, as well as a growing understanding of IgG4-related diseases. The demographic profile of our cohort is strongly consistent with established epidemiological data. The average age at diagnosis is typically between 40 and 60 years. There is a documented male predominance, with a male-to-female ratio of 2:1 to 3:1. In our series, the mean age was 54 years. Our observed male-to-female ratio was precisely 3:1 (18 men and six women), consistent with the characteristic demographic profile of this rare disorder.

The pathophysiology of idiopathic and secondary retroperitoneal fibrosis remains incompletely understood. Several theories have been proposed to explain the progression of this pathological process. However, it appears that a combination of immunological, genetic, and atherosclerotic factors contributes to the retroperitoneal inflammatory and fibrotic response [7,8]. Plasma cell overexpression of IgG4 and elevated serum IgG4 levels have been described in RPF [8,9]. RPF is idiopathic in approximately 70% of cases, whereas secondary forms account for nearly 30% [1,6,7].

Secondary RPF may result from medications, malignancies, infections, radiation therapy, trauma, abdominal surgery, retroperitoneal hemorrhage, local or systemic inflammatory disorders, or vasculitis [1]. In contemporary literature, idiopathic forms typically represent approximately 70% of cases, while secondary forms account for the remaining 30% [1,6,7]. The results of our study are consistent with these observations, as idiopathic RPF represented 75% of our cohort. Furthermore, modern classifications increasingly recognize the critical role of IgG4-related disease in idiopathic cases, a paradigm shift that makes direct statistical comparisons with older historical cohorts challenging.

Fibrosis usually progresses slowly, and some cases remain asymptomatic until compression of adjacent structures occurs. Consequently, retroperitoneal fibrosis may be discovered incidentally during imaging studies performed for unrelated reasons [10]. The symptoms of RPF are generally insidious, nonspecific, and highly variable. Pain remains the most common manifestation, reported in 80-92% of cases [11]. Urinary symptoms may mimic lower urinary tract disorders, whereas vascular manifestations can include intermittent claudication or unilateral/bilateral varicocele due to vascular compression [10,11].

The clinical presentation observed in our cohort is consistent with previous reports [10,11]. Low back pain was the predominant symptom, affecting 19 patients (79.2%), while constitutional manifestations were common, particularly weight loss in 17 patients (70.8%) and fever in seven patients (29.2%). Imaging plays a central role in the diagnosis of RPF. CECT and MRI provide reliable information regarding the extent and anatomical relationships of the fibrotic mass [2,3]. Although histological examination remains the diagnostic gold standard, biopsy is currently reserved mainly for cases in which a neoplastic, infectious, or other secondary cause is suspected [7].

**Figure 2:**
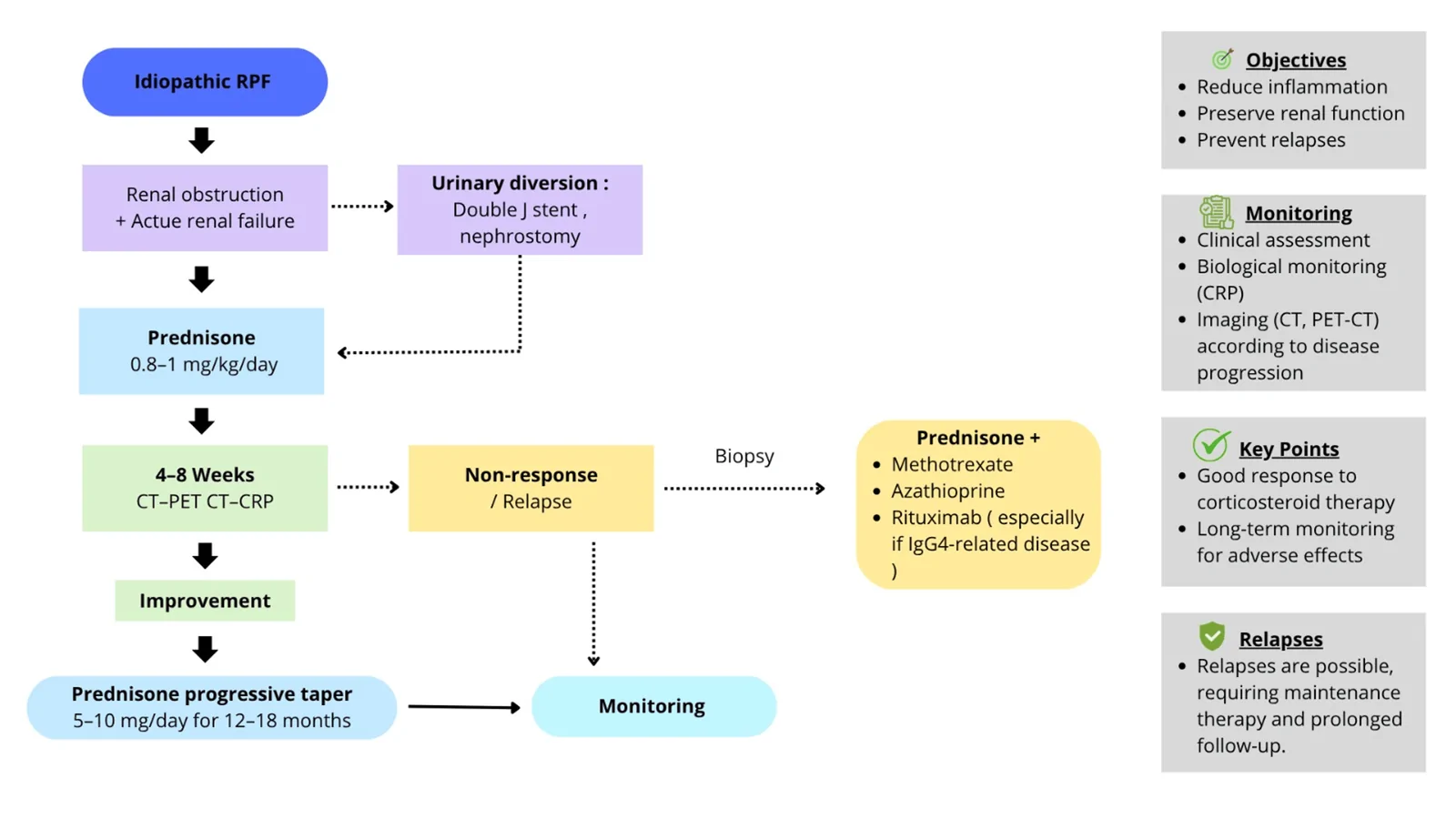
Management strategy for retroperitoneal fibrosis RPF: retroperitoneal fibrosis; CECT: contrast-enhanced computed tomography ; PET-CT: positron emission tomography-computed tomography; CRP: C-reactive protein; IgG4: immunoglobulin G4 The figure was designed by the authors using Canva® (Canva Pty Ltd., Sydney, Australia)

The therapeutic management of RPF primarily depends on the underlying etiology. Secondary forms require treatment of the causative condition, including discontinuation of offending medications, treatment of infections, surgical management, or oncological therapy when appropriate. In idiopathic forms, treatment remains largely empirical, and no universally accepted guidelines currently define the optimal therapeutic strategy [4,5]. To address this lack of universal guidelines, our university department has developed a standardized institutional management algorithm based on the available literature, which guided the therapeutic management of our cohort (Figure 2).

In clinical practice, management relies on three complementary approaches: emergency urinary drainage in patients presenting with obstructive uropathy or renal impairment, systemic corticosteroid therapy as the first-line medical treatment, and ureterolysis in selected patients with persistent ureteral obstruction despite medical therapy [12]. Prednisone is generally administered at a dose of 0.5-1 mg/kg/day for two to four weeks, followed by gradual tapering to a maintenance dose of approximately 10 mg/day [4].

Patients with RPF associated with abdominal aortic aneurysm require a distinct therapeutic approach. Initial management is based on vascular repair, combined with corticosteroid therapy when the fibrotic process persists [4]. Because prolonged corticosteroid therapy may lead to significant adverse effects, several steroid-sparing agents have been proposed, including azathioprine, mycophenolate mofetil, and tamoxifen, with outcomes comparable to those achieved with corticosteroids alone [7]. In cases of urinary obstruction associated with renal failure, urgent urinary diversion is required (Figure 2). Double-J ureteral stenting remains the most frequently used technique, although percutaneous nephrostomy may also be considered. In published series, approximately half of patients require emergency urinary drainage. Open surgery may occasionally be necessary, particularly when a tissue diagnosis is required [12,13].

Ureterolysis is currently reserved for refractory cases, especially when medical treatment fails to reduce the fibrotic mass or relieve ureteral obstruction. Owing to the predominantly fibrotic nature of persistent lesions, surgical intervention is generally considered only after prolonged medical therapy has failed [13,14]. The prognosis of RPF is generally favorable, with reported 10-year survival rates exceeding 70% [12,15,16]. Mortality is usually related to underlying malignancies or cardiovascular complications rather than to RPF itself [17].

Late recurrences remain unpredictable and may occur from several months to more than nine years after treatment. Recurrence is most common during the first five years of follow-up, supporting the need for prolonged clinical, biochemical, and radiological surveillance [17]. In our cohort, relapse occurred in four patients (16.7%) after a median follow-up period of three years (range: 12 months to five years), highlighting the importance of long-term monitoring even after initial clinical improvement. In our series, all patients underwent urinary drainage with double-J stent placement and received systemic corticosteroid therapy. Renal function improved in 40% of the patients who presented with acute renal failure (6/15) following treatment, and no deaths directly attributable to retroperitoneal fibrosis or its treatment were recorded.

Limitations

This study has several limitations. First, its retrospective design inherently introduces risks of selection bias, information bias, and potential outcome misclassification. Second, as a single-center study conducted in a tertiary university hospital, our cohort is subject to referral and spectrum bias; we likely manage more severe or complicated cases, meaning that these results may not be fully generalizable to broader or lower-acuity populations. Third, the lack of routine serum IgG4 assessment and specific immunostaining in our earlier cases limits our ability to retrospectively classify patients with idiopathic disease according to modern IgG4-related disease criteria. Fourth, the follow-up duration was heterogeneous across the cohort, with some patients having incomplete long-term data, which may lead to an underestimation of late recurrences. Finally, the descriptive nature of this case series and the absence of a comparator or control group restrict our ability to draw definitive conclusions regarding the superiority of our therapeutic approach.

## Conclusions

This retrospective series highlights that RPF is a rare but potentially functionally threatening condition. In our institutional experience, a standardized management strategy combining prompt urinary drainage with corticosteroid therapy was associated with favorable clinical outcomes, including renal function improvement in 40% of the patients who initially presented with acute renal failure. However, given the descriptive nature of this study, the lack of a control group, and the inherent limitations of retrospective data, these findings cannot definitively establish a causal relationship between treatment and clinical outcomes. Further multicenter, prospective studies with standardized comparator groups and modern immunologic profiling remain essential to optimize and individualize the therapeutic approach to this complex disease.
